# Study of matrix metalloproteinases and their inhibitors in prostate cancer

**DOI:** 10.1038/sj.bjc.6605569

**Published:** 2010-02-16

**Authors:** S Escaff, J M Fernández, L O González, A Suárez, S González-Reyes, J M González, F J Vizoso

**Affiliations:** 1 Servicio Urología, Hospital de Jove, Gijón, Spain; 2 Unidad de Investigación, Hospital de Jove, Gijón, Spain; 3 Servicio de Urología, Hospital Universitario Central de Asturias, Spain; 4 Unidad Multidisciplinario de Investigación en Oncología Quirúrgica del Instituto Universitario de Oncología del Principado de Asturias, Oviedo, Spain

**Keywords:** prostate carcinoma, tissue arrays, prognosis, MMP, TIMP, PSA

## Abstract

**Background:**

Extracellular matrix metalloproteases (MMPs) have raised an extraordinary interest in cancer research because of their potential role in basal membrane and extracellular matrix degradation, consequently facilitating tumour invasion and metastases development.

**Methods:**

An immunohistochemical study was performed using tissue arrays and specific antibodies against MMPs 1, 2, 7, 9, 11, 13, 14, and their tissue inhibitors, TIMPs 1, 2 and 3. More than 2600 determinations on cancer specimens from 133 patients with clinically localised prostate carcinoma, 20 patients with prostatic intraepithelial neoplasia and 50 patients with benign prostate hyperplasia and controls, were performed.

**Results:**

When compared with benign pathologies, prostate carcinomas had higher expression of all MMPs and TIMPs. Dendogram shows a first-order division of tumours into two distinct MMPs/TIMPs molecular profiles, one of them with high MMPs/TIMs expression profile (*n*=70; 52.6%). Tumours with high expression of MMP-11 or -13, or cluster thereof, were significantly associated with higher probability of biochemical recurrence.

**Conclusion:**

The expression of MMPs and TIMPs seems to have an important role in the molecular biology of prostate carcinomas, and their expression by tumours may be of clinical interest to used as indicators of tumour aggressiveness.

The prevalence of prostate cancer is so high that it could be considered as a normal age-related phenomenon (Hughes *et al*, 2005). Several published autopsy series have shown that up to one-third of men between the ages of 30 and 40 years harbour histological evidence of prostate carcinoma (Sakr *et al*, 1994).

A significant minority of patients undergoing radical prostatectomy for clinical organ-confined disease will ultimately be found to have pathological evidence of spread outside the prostate. Although these patients may be expected to have progression and survival rates comparable to those of patients with clinical advanced clinical disease, as defined by grade and serum prostate-specific antigen (PSA) level, those men who present with clinical stage T3 are likely to have greater tumour volume, higher grade and increased likelihood of regional spread. Currently, the majority of men undergoing prostatectomy for pathologically advanced disease are categorised as high risk on the basis of serum PSA value or biopsy Gleason score. Nevertheless, there is some overlap in the groups of men undergoing radical prostatectomy for clinical stage T3 and for pathological stage T3 (Meng and Carrol, 2007).

Despite recent improvement in diagnostic and therapeutic techniques, the survival rate of prostate cancer patients remains poor due to post-treatment recurrence disease. Despite all the recent efforts in the identification of molecular mechanisms involved in the progression of prostate cancer, tumour progression in the prostatic compartment, as well as in the metastasis compartment, is poorly understood (Logothetis and Lin, 2005). These pitfalls underscore the need for new risk markers that allow the early detection of carcinogenesis and, therefore, of cancer relapse.

Degradation of the stromal connective tissue and basement membrane components are key elements in tumour invasion and metastasis. This is particularly true with the interstitial collagens, which are very resistant to proteolytic attacks, being degraded only by matrix metalloproteinases (MMPs) (Nelson *et al*, 2000). The human MMP family currently consists of 28 members of homologous zinc-dependent endopeptidases that can be divided into eight structural classes or, on the basis of their substrate specificity and primary structure, into the more familiar subgroups of collagenases (MMP-1, -8 and -13), gelatinases (MMP-2 and -9), stromelysins (MMP-3, -10, -11), membrane-associated MMPs (MMP-14, -15, -16, -17, -23, -24, -25) and other novel MMPs (Brinckerhoff *et al*, 2000; Overall and Lopez-Otin, 2002; Demers *et al*, 2005). The MMPs are synthesised as inactive zymogens, which are then predominantly activated pericellularly by either other MMPs or by serine proteases. On the other hand, there are available data clearly challenging the classic dogma stating that MMPs promote metastases exclusively by modulating the remodelling of extracellular matrix. Indeed, MMPs have been identified that are able to affect *in vivo* tumour cell behaviour as a consequence of their ability to cleave growth factors, cell surface receptors, cell adhesion molecules or chemokines/cytoquines (Manes *et al*, 1999; Noe *et al*, 2001; Egeblad and Werb, 2002; Turk *et al*, 2004). Furthermore, by cleaving proapoptotic factors, MMPs are able to produce a more aggressive phenotype through generation of apoptotic resistant cells (Fingleton *et al*, 2001). The MMPs may also regulate cancer/related angiogenesis, both positively through their ability to mobilise or activate proangiogenic factors (Stetler-Stevenson, 1999), and negatively through generation of angiogenesis inhibitors, such as angiostatin and endostatin, which are cleaved from large protein precursors (Cornelius *et al*, 1998). The MMPs' activities are specifically inhibited by the so-called tissue inhibitors of metalloproteases (TIMPs). Currently, four different TIMPs are known to exist: TIMPs 1, 2, 3 and 4. However, it is now assumed that TIMPs are multifactorial proteins that are also involved in the induction of proliferation and inhibition of apoptosis (Wurtz *et al*, 2005).

The expression in prostate cancer of several MMPs and TIMPs, such as MMP-2, -7, -9, -13 and -14, TIMP-1, -2 and -3, has been recently reported (Brehmer *et al*, 2003; Pang *et al*, 2004; Zhang *et al*, 2004; Morgia *et al*, 2005; Riddick *et al*, 2005; Semaan *et al*, 2005; Cardillo *et al*, 2006). In addition, recent studies have shown that overexpression of MMPs induces prostate tumour growth and increases the development of metastasis (Bratland *et al*, 2003; Daja *et al*, 2003; Cao *et al*, 2005, 2008; Dong *et al*, 2005; Lynch *et al*, 2005; Nabha *et al*, 2006; Bonfil *et al*, 2007; Millimaggi *et al*, 2007; Pulukuri and Rao, 2008). Likewise, an association between MMPs and/or TIMPs expression and parameters indicative of tumoural aggressiveness or poor outcome in patients with prostate cancer has also been reported (Gohji *et al*, 1998; Brehmer *et al*, 2003; Trudel *et al*, 2003; Morgia *et al*, 2005; Riddick *et al*, 2005; Semaan *et al*, 2005; Cardillo *et al*, 2006).

The objectives of this study were to evaluate the expression and clinical relevance of several MMPs and TIMPs of previously recognised biological importance in prostate carcinomas, using the tissue array (TA) technique. This technique has allowed us the processing of a large number of tissue specimens for a wide range of protein determinations.

## Materials and methods

### Patients and tissues samples

The histological material used in this study was obtained from 133 patients with clinical localised prostate carcinoma (age range 44–79 years), from 20 patients with prostatic intraepithelial neoplasia (PIN) (age range 54–70 years) and from 50 patients with benign prostate hyperplasia (BPH) (age range 54–70 years). We selected patients with prostate adenocarcinomas that had undergone radical retropubic prostatectomy and had a minimum of 5-year follow-up in those cases that did not present a biochemical recurrence. The exclusion criteria were the following: metastatic disease at presentation, previous history of any type of malignant tumour, having received any type of neoadjuvant therapy, development of a second primary cancer and absence of sufficient tissue in the paraffin blocks used for manufacturing TAs. From a total of 158 patients fulfilling these criteria, we selected randomly a sample size of 133 patients, divided in two different groups of similar size and stratified with regard to the development of biochemical recurrence, which was the key variable of the study. Of these patients, 47 presented biochemical recurrence (PSA level >0.2 ng ml^–1^, with a second confirmatory determination).Patients and tumour characteristics are listed in Table 1. Tumours were staged according to the 1992 TNM classification. (Flemming *et al*, 1997). Histological tumour grading was established according to the Gleason criteria (Whitmore, 1956). The PSA serum levels were determined, preoperatively and postoperatively, using the ‘Elecys’ immune-assay tests (Roche Diagnostic GmbH, Mannheim, Germany). Determination of PSA serum levels was performed 1 month after surgical treatment, finding undetectable levels in all patients. Finally, all cases were evaluated for disease recurrence or survival status by clinical, radiological and biological examinations every 6 months. The mean follow-up period was 62 months (range: 6–144 months). Patients were treated according to the guidelines used in our institutions. The study adhered to national regulations and was approved by our institution's Ethics and Investigation Committee.

### Tissue arrays and immunohistochemistry

All radical retropubic prostatectomy specimens were routinely fixed in 10% neutral-buffered formalin and stored after being embedded in paraffin at room temperature from 4 months to 5 years before further testing was performed. Histopathological representative tumour areas were defined on haematoxylin and eosin-stained sections and marked on the slide. Tumour TA blocks were obtained by punching a tissue cylinder (core) with a diameter of 1.5 mm through a histological representative area of each ‘donor’ tumour block, which was then inserted into an empty ‘recipient’ TA paraffin block using a manual tissue arrayer (Beecher Instruments, Sun Prairie, WI, USA) as described elsewhere (Parker *et al*, 2002). Collection of tissue cores was carried out under highly controlled conditions. Two cores were used for each case.

Four composite high-density TA blocks were designed, and serial 5-*μ*m sections were consecutively cut with a microtome (Leica Microsystems GmbH, Wetzlar, Germany) and transferred to adhesive-coated slides. One section from each TA block was stained with haematoxylin and eosin, and these slides were then reviewed to confirm that the sample was representative of the original tumour. Immunohistochemistry was carried out on these sections of TA fixed in 10% buffered formalin and embedded in paraffin using a TechMate TM50 autostainer (Dako, Glostrup, Denmark). Antibodies for MMPs and TIMPs were obtained from Neomarker (Lab Vision Corporation, Fremont, CA, USA). The dilution for each antibody was established based on negative and positive controls (1/50 for MMP-2, -7 and -14, and TIMP-2 and -3; 1/100 for MMP-1, -9 and –13, and TIMP-1; and 1/200 for MMP-11).

Tissue sections were deparaffinised in xylene, and then rehydrated in graded concentrations of ethyl alcohol (100, 96, 80, 70%, then water). To enhance antigen retrieval only for some antibodies, TA sections were microwave-treated (H2800 Microwave Processor, EBSciences, East Granby, CT, USA) in citrate buffer (Target Retrieval Solution, Dako) at 99°C for 16 min. Endogenous peroxidase activity was blocked by incubating the slides in peroxidase-blocking solution (Dako) for 5 min. The EnVision Detection Kit (Dako) was used as the staining detection system. Sections were counterstained with haematoxylin, dehydrated with ethanol and permanently coverslipped.

### Tissue arrays analysis

For each antibody preparation studied, the location of immunoreactivity, percentage of stained cells and intensity were determined. All the cases were semiquantified for each protein-stained area. An image analysis system with the Olympus (Münster, Germany) BX51 microscope and analysis soft (analySIS, Soft imaging system, Münster, Germany) was used as follows: tumour sections were stained with antibodies according to the method explained above and counterstained with haematoxylin. There are different optical thresholds for both stains. Each core was scanned with a × 400-power objective in two fields per core. Fields were selected searching for the protein-stained areas. The computer program selects and traces a line around antibody-stained areas (red spots correspond to higher optical thresholds), with the remaining, non-stained areas (haematoxylin-stained tissue with lower optical threshold) standing out as a blue background. Any field has an area ratio of stained (red) *vs* non-stained areas (blue). A final area ratio was obtained after averaging two fields. To evaluate immunostaining intensity, we used a numerical score ranging from 0 to 3, reflecting the intensity as follows: 0, no staining; 1, weak staining; 2, moderate staining; and 3, intense staining. Using an Excel spreadsheet, the mean score was obtained by multiplying the intensity score (I) by the percentage of stained cells (Liu *et al*, 2001) and the results were added together (total score: I × PC). This overall score was then averaged with the number of cores that were done for each patient. If there was no tumour in a particular core, then no score was given. In addition, for each tumour, the mean score of two core biopsies was calculated.

Furthermore, whole-tissue sections from blocks from a subset of ten cases for either tumour, PINs or BPH specimens, were compared with the corresponding TA discs, regarding each MMP and TIMP expression. Those cases were selected randomly, and the obtained clinicopathological data were very similar to those from the whole series. Each whole-tissue section was scanned with a × 400-power lens in ten different fields. Fields were selected searching for the protein-stained areas, such as it was described above.

### Data analysis and statistical methods

Differences in percentages were calculated with the *χ*^2^-test. Immunostaining score values for each protein were expressed as median (range). Comparison of immunostaining values between groups was made with the Mann–Whitney or Kruskal–Wallis tests. For metastasis-free survival analysis, we used the Cox univariate method. Cox regression model was used to examine interactions of different prognostic factors in a multivariate analysis. Expression profiles were analysed by unsupervised hierarchical clustering method that organises proteins in a tree structure, based on their similarity. Data were reformatted as follows: –‘3’ designated negative staining, ‘3’ positive staining, missing data were left blank. The score values were reformatted (positive-negative) choosing the median as cutoff value. We used the Cluster 3.0 program (average linkage, Pearson's correlation). Results were displayed with Treeview (Eisen *et al*, 1998). The SPSS 17.0 software was used for all calculations (SPSS Inc., Chicago, IL, USA).

## Results

More than 2600 determinations were performed on TAs from 133 patients with clinically localised prostate carcinoma, from 20 patients with PIN, from 50 patients with BPH specimens and controls. Minimal internal variance of score data between duplicate tissue cores from the same patients was detected in TAs, showing a high agreement for each protein (*r* >0.95 and *P*<0.0001, for each protein). In the validation study, there was a total concordance in the global expression, as well as in the intensity of immunostaining, for each MMP and TIMP between TAs and the corresponding whole-tissue sections. In addition, there were highly significant correlations in the immunostaining scores between these two-paired sets (*r* >0.90 and *P*<0.0001, for each protein).

**Figure 1:**
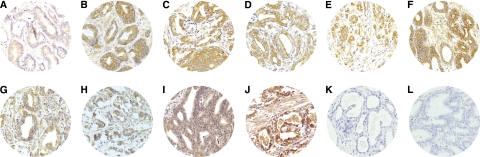
Examples of tissue arrays (TAs) with immunostaining for each protein evaluated in prostate carcinoma. (**A**) Matrix metalloproteinase (MMP)-1, (**B**) MMP-2, (**C**) MMP-7, (**D**) MMP-9, (**E**) MMP-11, (**F**) MMP-13, (**G**) MMP-14, (**H**) tissue inhibitors of metalloprotease (TIMP)-1, (**I**) TIMP-2, (**J**) TIMP-3, (**K**) normal tissue and (**L**) tumour with IgG.

**Figure 2:**
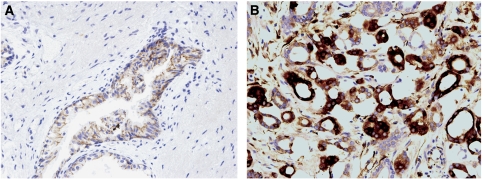
(**A**) Example of immunostaining for matrix metalloproteinase (MMP)-14 in prostate benign pathology. Magnification: × 200. (**B**) Example of immunostaining for MMP-14 in prostate carcinoma. Magnification: × 200.

Figure 1 shows some examples of TAs with immunostaining for each protein evaluated. Immunostaining for all the proteins studied was localised predominantly in tumour cells, but also in stromal cells of a significant percentage of prostate carcinomas. However, immunostaining for all the proteins studied was predominantly localised in epithelial cells when prostate benign pathologies were analysed (Figure 2).

As Table 2 shows, we first compared the crude expression of score values for MMPs and TIMPs between PINs, HBPs and prostate carcinomas. Prostate carcinomas had higher expression of all MMPs and TIMPs compared with benign pathologies.

It is also noteworthy that there was a wide variability in the immunostaining score values for each protein in prostate carcinomas, which spread more widely than those score values in benign pathologies (Table 2).

We also evaluated the possible relationship between MMPs and TIMPs expressions and clinicopathological factors of prostate carcinomas, such as age of patients, tumour stage, histological grade and pre-treatment serum levels of PSA. Our results only showed significant association of TIMP-1 with histological grade. Thus, tumours with higher score values for TIMP-1 had a higher percentage of cases with high Gleason score (score 2–4: (number of cases (percentage) 14 (21.2); score 5–6: 31 (47); score 7–10: 21 (31.8)) compared to tumours with lower score values for TIMP-1 (score 2–4: 4(6); score 5–6 (53.7); score 7–10: 27 (40.31)) (*P*=0.036).

**Figure 3:**
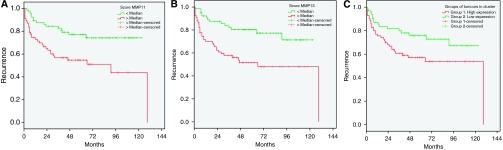
Probability of biochemical recurrence as function of score values for matrix metalloproteinase (MMP)-11 (**A**), score values for MMP-13 (**B**) and as function of the two mayor clusters of tumours (Groups 1 and 2) (**C**). Median value of score values was chosen as cutoff value.

With regard to outcome from patients with prostate carcinomas, our results showed a significant association between score values of MMP-11 or -13 and biochemical recurrence. Patients with tumours showing MMP-11 or -13 score values greater than median had a significant higher probability of biochemical recurrence than those patients with lower MMP-11 or -13 score values (*P*=0.02 and *P*=0.001, respectively) (Figures 3A and B, respectively).

**Figure 4:**
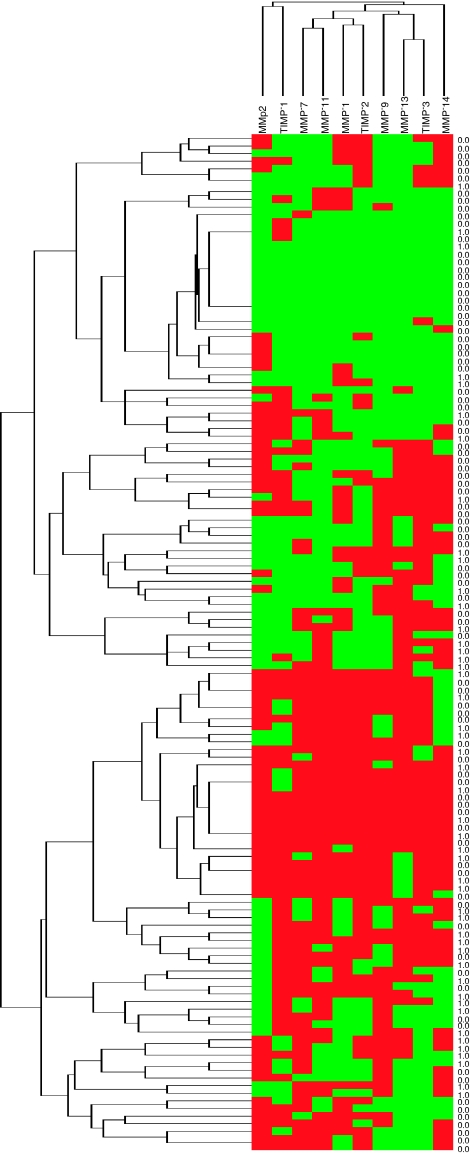
Graphical representation of two-dimensional unsupervised hierarchical clustering results based on immunohistochemistry expression profiles of 10 proteins in 150 prostate carcinoma samples. Rows: samples; columns: proteins. Protein expression scores are depicted according to a colour scale: red: positive staining; green: negative staining; grey: missing data. Dendogram of samples (to the left of matrix) and proteins (above matrix) represent overall similarities in expression profiles. The status column: 1=with recurrence; 0=without recurrence, at the census point. Two major clusters of tumours (1 and 2) are shown for score values and in tumoural cells. The colour reproduction of the figure is avilable on the html full text version of the paper.

In addition, to identify specific groups of tumours with distinct MMP/TIMP expression profiles the data were analysed by unsupervised hierarchical cluster analysis. The algorithm orders proteins on the horizontal axis and samples on the vertical axis based on similarity of their expression profiles. When we dichotomised cases with regard to their score values as using the median value for each MMP or TIMPs as cutoff point were considered, a dendogram showing a first-order division of the tumours into two distinct MMP/TIMP molecular profiles were obtained. In this way, one of them was designated as group 1 with a high MMPs/TIMPs expression profile (*n*=70) and the other was designed as group 2 (*n*=63) with a low MMPs/TIMPs expression profile (Figure 4). In addition, our results showed significant differences in prognosis between the two groups of patients corresponding to these two types of tumours, corresponding cases with high MMPs/TIMPs expression profile tumours with those patients with higher risk of biochemical recurrence (Table 3 and Figure 3C).

Multivariate analysis according to Cox model showed that tumour stage (PT 3–4: relative risk, RR=3.38; 95% confidence interval, CI=1.7–6.5; *P*<0.0001) and Gleason grading 7–10: 2.08 (1.1–3.9); *P*<0.05) were significantly and independently associated with biochemical recurrence. However, this same analysis also showed that expressions of MMP-13, as well as clustering for score values, were also independent factors associated with biochemical recurrence in patients with prostate cancer (Table 3).

## Discussion

Our results clearly showed higher MMPs and TIMPs expressions in prostate carcinomas than either in PIN or in BPH, which seems to reflect an important mechanism in the molecular biology of prostate cancer. Similarly, some authors found significantly higher expressions of MMP-1, -2 and -9 in prostate cancer tissues than in BPH tissues (Zhong *et al*, 2008). This seems to indicate that high expressions of MMPs and TIMPs might identify prostate benign lesions with risk to develop cancer or even associated to undetected malignant lesions. Therefore, these findings could be of importance with regard to design preventive strategies and/or for further studies of prostate cancer prevention based on enzymatic inhibition of the MMPs/TIMPS system. Our finding that stromal expression was found in cancer but not in BPH, where the MMPs were localised in glandular epithelial cells, was also especially remarkable. We speculate that these findings may be due to epithelial–mesenchymal transition. In this way, mesenchymal cells may again acquire a differentiated epithelial phenotype through a mesenchymal-to-epithelial transition, which might mean in terms of the metastatic process.

Our data also support the biological heterogeneity of prostate carcinomas regarding the expressions of these parameters implicated in tumour invasion and metastasis. The MMPs are implicated in basic processes of tumour progression, such as degradation of basement membrane and extracellular matrix, stimulation of cellular proliferation, cellular motility, resistance to apoptosis and angiogenesis. Therefore, the diverse clinical evolution of prostate tumours may depend their expressions of MMPs and TIMPs. Our results indicating an association between TIMP-1 expression and higher tumour grade were especially remarkable. This suggests that TIMP-1 is associated with aggressive behaviour in prostate carcinomas. If TIMPs inhibit MMPs *in vivo*, it should be expected that high levels of these inhibitors would prevent tumour progression and thus be related with low aggressiveness of tumours. However, TIMPs are multifunctional proteins that, in addition to its MMP-inhibitory effect, also show distinct tumour-stimulatory functions involved in the induction of proliferation and inhibition of apoptosis (Jiang *et al*, 2002; Wurtz *et al*, 2005).

In this study, we also investigated the possible relationship between each one of MMPs or TIMPs expressions and clinical outcome, such as PSA-defined recurrence after radical prostatectomy in our studied population. Our results showed that the global expression of MMP-11 and -13 by prostate carcinomas correlated with higher incidence rate of biochemical relapse. Therefore, the global expression of MMP-11 and -13 by prostate carcinomas may, in combination with other factors, support useful prognostic information for a more optimal follow-up and treatment from these patients. Likewise, our data led us to consider that MMP-13 and/or MMP-11 may be optimal therapeutic targets for inhibition in prostate carcinoma. These results are in accordance with previous studies that associated expressions of MMP-11 or -13 with poor prognostic in other tumours, such as breast cancer (Gonzalez *et al*, 2007; Vizoso *et al*, 2007).

The MMP-13 (collagenase-3) has been found to have an exceptionally wide substrate specificity when compared with other MMPs (Freije *et al*, 1994; Knauper *et al*, 1997). Moreover, it is thought to have a central role in the MMP activation cascade, both activating and being activated by several other MMPs (MMP-14, -2 or -3). The MMP-13 has been detected to be expressed by different prostate cancer cell lines, prostate cancer tissue and BPH (Varani *et al*, 2001; DeClerck *et al*, 2004; Pang *et al*, 2004). Its expression pattern by prostate cells seemed to be varied according to the malignancy of prostatic cells and, therefore, it has been suggested to be a diagnostic marker for prostate cancer (Morgia *et al*, 2005). In addition, recent report indicated that androgen acts to stimulate the expression level of MMP-13 by LNCaP prostate cancer cell line (Pang *et al*, 2004). It has also been showed that plasma concentrations of MMP-13 were high in patients with metastasis of prostate cancer, and in these patients decreased markedly after the therapy began (Morgia *et al*, 2005).

The MMP-11 (stromalysin-3) is preferential expressed by peritumour stromal cells (Basset *et al*, 1990, 1997) and high levels of MMP-11 were associated with tumour progression and poor prognosis in breast cancer (Chenard *et al*, 1996; Ahmad *et al*, 1998). However, at present there are few data on their expression and clinical signification in prostate cancer. The MMP-11 is a protease that can modulate cancer progression by remodelling extracellular matrix. It cleaves *α*1-antitripsin and IGF-BP1 (Remacle *et al*, 2000). Normal MMP-11 expression is present during embryogenesis and wound healing, and its expression in stressed epithelial cells is detected in the vicinity of fibroblasts (Boulay *et al*, 2001). The MMP-11 expression is observed in the area that surrounds malignant epithelial tumour cells and sometimes in tumour cells of oesophageal, oral, papillary thyroid, colorectal, skin and ovarian carcinomas (Thewes *et al*, 1999; Mueller *et al*, 2000; Soni *et al*, 2003; Wasenius *et al*, 2003; Yamashita *et al*, 2004). Hence, MMP-11 gene expression seems to be associated with tumour progression (Basset *et al*, 1997).

On the other hand, our data also show that it is possible to identify two phenotypes of prostate carcinomas with regard to their global expressions of MMPs/TIMPs. One of these two groups had high MMPs/TIMPs expression profile (*n*=70) and another designed group 2 (*n*=63) with low MMPs/TIMPs expression profile. Likewise, we found a significant relationship between these phenotypes of prostate carcinomas and biochemical recurrence. Thus, this classification may be relevant in relation to possible further therapies based on MMPs inhibition.

In summary, we found that MMPs/TIMPs expressions were in general higher in prostate carcinomas than in prostate benign tissues, which reflect an important role of these factors in the molecular biology of prostate carcinomas. In addition, there is variability in MMPs/TIMPs expressions in prostate carcinomas, which support the biological heterogeneity of these tumours. In addition, the expression of some MMPs and correlated significantly with prognosis. Thus, our results led us to consider that further studies on MMPs/TIMPs expressions may contribute to understand the biological and clinical behaviour of prostate carcinomas. In this way, further studies are needed to investigate differential profiles of MMPs/TIMPs expressions in patients with advanced prostate cancer that progress to hormone-refractory prostate cancer in spite of androgen-deprivation therapy.

## Figures and Tables

**Table 1:** Basal characteristics of 133 patients with prostate carcinoma

	**Without biochemical recurrence (*n*=86)**	**With biochemical recurrence (*n*=47)**	
**Characteristics**	**No. (%)**	**No. (%)**	***P*-value**
*Age (year)*			NS
<65	52 (39.5)	28 (40.4)	
>65	34 (60.5)	19 (59.6)	
			
*Tumoural stage*			<0.0001
T2	79 (91.9)	27 (57.4)	
T3–4	7 (8.1)	20 (42.6)	
			
*Gleason grading*			<0.003
2–4	14 (16.3)	4 (8.5)	
5–6	50 (58.1)	17 (36.2)	
7–10	22 (25.6)	26 (55.3)	
			
*PSA (ng ml*^*–1*^)			NS
<10	65 (75.6)	28 (59.6)	
>10	21 (24.4)	19 (40.4)	

Abbreviations: NS=not significant; PSA=prostate-specific antigen.

**Table 2:** Comparative analysis of the score values of expressions of metalloproteases and their inhibitors in benign prostate pathology and in prostate carcinomas

	**Score values (median (range))**			
**Factor**	**PIN (*n*=20)**	**BPH (*n*=50)**	**Prostate cancer (*n*=133)**	***P*-value**
MMP-1	0 (0–47.9)	0 (0–62.43)	104.5 (0–245.1)	*P*<0.001
MMP-2	0 (0–32.6)	0 (0–44.6)	42.7 (0–239.6)	*P*<0.001
MMP-7	43.76 (0–61.4)	45.44 (0–75.7)	52.75 (0–235.5)	*P*<0.001
MMP-9	0 (0–25.84)	0 (0–24.96)	39.33 (0–123.9)	*P*=0.003
MMP-11	27.27 (0–50.18)	0 (0–68.6)	111.15 (0–275.5)	*P*<0.001
MMP-13	0 (0–40)	0 (0–50.23)	42.2 (0–151.7)	*P*<0.001
MMP-14	0 (0–39.06)	35.46 (0–89.35)	32.9 (0–290)	*P*<0.001
TIMP-1	0 (0–56.32)	0 (0–54.78)	33.9 (0–120.8)	*P*<0.001
TIMP-2	0 (0–43.7)	0 (0–41.87)	52.16 (0–238.8)	*P*<0.001
TIMP-3	24.86 (0–53.45)	28.61 (0–60.95)	43.35 (0–238.76)	*P*<0.001

Abbreviatons: BPH=benign prostatic hyperplasia; MMP=matrix metalloproteinase; PIN=prostatic intraepithelial neoplasm; TIMP=tissue inhibitors of metalloprotease.

**Table 3:** Cox univariate (HR) and multivariate (RR) analysis of the relationship between MMPs and TIMPs expression and relapse-free survival

**Factor**	**Event frequency**	**HR (95% CI)**	**RR (95% CI)**
MMP-1	21/26	1.1 (0.6–2)	1.2 (0.7–2.2)
MMP-2	28/19	0.6 (0.3–1)	0.6 (0.3–1.2)
MMP-7	18/29	1.7 (0.9–3)	1.3 (0.7–2.3)
MMP-9	19/28	1.6 (0.9–2.9)	1.2 (0.6–2.2)
MMP-11	15/32	2.5 (1.3–4.7)^**^	1.8 (0.9–3.4)
MMP-13	14/33	2.7 (1.4–5.2)^***^	2.6 (1.4–5)^**^
MMP-14	18/29	1.6 (0.9–2.9)	1.5 (0.8–2.7)
TIMP-1	20/27	1.5 (0.8–2.8)	1.3 (0.7–2.5)
TIMP-2	19/28	1.5 (0.8–2.8)	1.1 (0.6–2.1)
TIMP-3	21/26	1.3 (0.7–2.3)	1.2 (0.6–2.2)
Cluster 1 *vs* Cluster 2	16/31	1.9 (1–3.5)^*^	1.7 (0.9–3.2)^*^

Abbreviations: CI=confidence interval; HR=hazard ratio; MMP=matrix metalloproteinase; RR=relative risk; TIMP=tissue inhibitors of metalloprotease.

^**^P<0.005; ^***^P<0.001; ^*^P<0.05.
